# Identification and Gene Expression Analysis of a Taxonomically Restricted Cysteine-Rich Protein Family in Reef-Building Corals

**DOI:** 10.1371/journal.pone.0004865

**Published:** 2009-03-13

**Authors:** Shinichi Sunagawa, Michael K. DeSalvo, Christian R. Voolstra, Alejandro Reyes-Bermudez, Mónica Medina

**Affiliations:** School of Natural Sciences, University of California Merced, Merced, California, United States of America; American Museum of Natural History, United States of America

## Abstract

The amount of genomic sequence information continues to grow at an exponential rate, while the identification and characterization of genes without known homologs remains a major challenge. For non-model organisms with limited resources for manipulative studies, high-throughput transcriptomic data combined with bioinformatics methods provide a powerful approach to obtain initial insights into the function of unknown genes. In this study, we report the identification and characterization of a novel family of putatively secreted, small, cysteine-rich proteins herein named Small Cysteine-Rich Proteins (SCRiPs). Their discovery in expressed sequence tag (EST) libraries from the coral *Montastraea faveolata* required the performance of an iterative search strategy based on BLAST and Hidden-Markov-Model algorithms. While a discernible homolog could neither be identified in the genome of the sea anemone *Nematostella vectensis*, nor in a large EST dataset from the symbiotic sea anemone *Aiptasia pallida*, we identified SCRiP sequences in multiple scleractinian coral species. Therefore, we postulate that this gene family is an example of lineage-specific gene expansion in reef-building corals. Previously published gene expression microarray data suggest that a sub-group of SCRiPs is highly responsive to thermal stress. Furthermore, data from microarray experiments investigating developmental gene expression in the coral *Acropora millepora* suggest that different SCRiPs may play distinct roles in the development of corals. The function of these proteins remains to be elucidated, but our results from *in silico*, transcriptomic, and phylogenetic analyses provide initial insights into the evolution of SCRiPs, a novel, taxonomically restricted gene family that may be responsible for a lineage-specific trait in scleractinian corals.

## Introduction

The genome biology of basal metazoan phyla, such as the Cnidaria, promises to provide great insight into animal evolution. In terms of genomic resources, the hexacorallian lineage currently provides the largest dataset among the anthozoan cnidarians [1]. These resources include a draft genome of the sea anemone *Nematostella vectensis* [2], and expressed sequence tag (EST) datasets from the sea anemone *Aiptasia pallida* [3] and the reef-building corals *Acropora millepora* [4], *Acropora palmata*, and *Montastraea faveolata* [5]. Recent comparative studies have demonstrated that the genome of *N. vectensis* displays an “ancient complexity”, which was found to be more similar to vertebrates than to flies or nematodes despite its basal phylogenetic position [2], [6]. Similarly, the absence of key genes involved in innate immunity in the hydrozoan *Hydra magnipapillata*, but their presence in hexacorallians and sponges suggests that gene losses have occurred during the evolution of cnidarians [7].

Hexacorallians include stony corals (order Scleractinia), which commonly maintain a stable symbiosis with unicellular algae in the genus *Symbiodinium* and have the ability to build complex skeletal structures of calcium carbonate. Sea anemones (order Actiniaria) may also form symbioses with *Symbiodinium* spp., but they do not calcify. The discovery of genes that are found in only one or a limited number of taxa is of particular interest, since they may code for proteins responsible for taxon-specific adaptations [8].

Schwarz et al. (2008) reported a number of genes that appear to be limited to reef-building corals. Although bioinformatics methods may help discovering such candidate genes through homology searches, an intrinsic challenge that remains is their functional characterization. As a systematic approach, a correlation with expression patterns of known genes under controlled experimental conditions may suggest a similar physiological role of such genes, or at least, define conditions under which their expression is responsive. Microarray-based transcriptome analyses allow for assaying thousands of genes in a single experiment [9] and have recently become available for scleractinian corals [10], [11]. In particular, the application of clustering methods to group genes with similar expression patterns provides a powerful tool to organize and identify functionally related genes and their networks [12].

In the present study, we report the identification and characterization of a family of small, cysteine-rich proteins (SCRiPs) by data mining EST libraries constructed from reef-building corals. Homology searches in currently available databases suggest that members of this family are limited to the scleractinian corals. Although the functions of these SCRiPs require further investigation, our gene expression microarray data analyses provide evidence that SCRiPs are responsive to thermal stress, and that different SCRiPs may have acquired distinct functions during their evolution.

## Results

### *In silico* identification of Small Cysteine-Rich Proteins (SCRiPs)

The initial search aimed at the discovery of antimicrobial peptides, in particular β-defensin-like peptides in a *Montastraea faveolata* EST collection [5]. For this purpose, we aligned the six-cysteine-motif containing domains of β-defensins (pfam00711) and constructed a first Hidden Markov Model (HMM). Querying the *M. faveolata* ESTs using this model resulted in the identification of one β-defensin-like protein sequence. We named this protein Small Cysteine-Rich Protein 1 (Mfav-SCRiP1). We subsequently used this novel sequence in a tBLASTn search against the EST library and discovered three additional sequences: Mfav-SCRiP2, Mfav-SCRiP3a, and Mfav-SCRiP4. Next, we constructed a second HMM using the aligned, cysteine-rich regions of Mfav-SCRiP1-4, and queried the model in a second round against the EST library. This search resulted in the identification of two more sequences: Mfav-SCRiP3b and Mfav-SCRiP5. All newly discovered candidates were used in a second tBLASTn search, which revealed the sequences Mfav-SCRiP6-8. Finally, a third HMM model containing all newly discovered SCRiP sequences was constructed and queried against the *M. faveolata* EST set without identifying additional sequences (the procedure is summarized in Table 1). Subsequent tBLASTn searches in the non-redundant nucleotide (nt) and EST (est_others) databases at NCBI matched homologous sequences in the coral species *Montipora capitata* (Mcap-SCRiP1a and 1b) and *Acropora millepora* (Amil-SCRiP1-3). It should be noted that the numbering of SCRiP sequences was based on the order of their identification and do not imply orthology.

**Table 1:** Summary of iterative search strategy used to identify Mfav-SCRiP sequences.

Step	Screening method	Query against	Sequences used	Identification of
1	HMM	sixframe-translated	Conserved domains of β-defensins (pfam00711)	Mfav-SCRiP1
		EST library sequences		
2	tBLASTn	EST library sequences	Mfav-SCRiP1	Mfav-SCRiP2
				Mfav-SCRiP3a^a^^,^ b
				Mfav-SCRiP4
3	HMM	sixframe-translated	Mfav-SCRiP1-4	Mfav-SCRiP3b^a^^,^ b
		EST library sequences		Mfav-SCRiP5
4	tBLASTn	EST library sequences	Mfav-SCRiP1-6	Mfav-SCRiP6
				Mfav-SCRiP7^b^
				Mfav-SCRiP8
5	HMM	sixframe-translated	Mfav-SCRiP1-8	no additional sequences
		EST library sequences		
6	tBLASTn	nt (NCBI), est_others (NCBI)	all Mfav-SCRiPs	Amil-SCRiP1
				Amil-SCRiP2
				Amil-SCRiP3
				Mcap-SCRiP1a
				Mcap-SCRiP1b

a likely to be a longer isoform of SCRiPs.

b possibly a pseudogene.

### Novelty of SCRiPs and their restriction to the order Scleractinia

Both BLASTp and tBLASTn searches (E-value cutoff: 0.01) of the protein sequences against the GenBank non-redundant protein and nucleotide databases (nr and nt, respectively) returned no significant hits other than the SCRiPs identified in this study. Remarkably, tBLASTn searches in the genome and EST databases of the sea anemone *Nematostella vectensis* (JGI: Nemve1.fasta, and Nv_ESTs_060207.fa, respectively), the closest relative to *M. faveolata* for which a genome draft is available, did not identify any discernable homologs either. Similarly, no homolog could be identified in an EST data set (>10,000 ESTs) from the symbiotic sea anemone *Aiptasia pallida* [3]. These results indicate that homologous proteins or conceptual nucleotide translations have not been reported yet. Also, querying a HMM of all identified SCRiPs against the non-redundant protein database (NCBI) resulted in no significant hits, which together with the BLAST search results supports the taxonomic restriction of this protein family to the order Scleractinia.

### Verification of Mfav-SCRiP transcripts

In order to verify that the new sequences represent true expressed transcripts we set the requirement that each SCRiP sequence must be supported by at least two independently sequenced cDNA clones. For Mfav-SCRiP1, Mfav-SCRiP4 and Mfav-SCRiP6, three or more cDNA clones from the EST library provided the data that were assembled to the reported consensus sequences. The two cDNA clones coding for Mfav-SCRiP3a and Mfav-SCRiP3b most likely originate from the same transcript, and may represent not completely spliced artifacts of Mfav-SCRiP2 (Figure S1). Alternatively, Mfav-SCRiP3a/b may represent a longer variant of SCRiPs or possibly a pseudogene. All other Mfav-SCRiPs were successfully re-sequenced in 5′ and 3′ RACE reactions. As a result, Mfav-SCRiP7 was excluded from further analysis, as its nucleotide sequence from the *M. faveolata* EST library (DR987965 and DR987964) showed a frame-shift mutation, which was confirmed by re-sequencing of two RACE clones (data not shown).

### Characterization of SCRiPs

**Figure 1:**
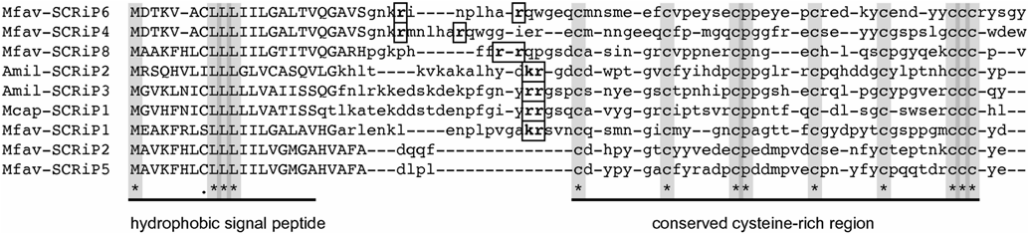
Multiple sequence alignments of SCRiPs identified in this study. Predicted signal peptides are shown in upper case and conserved amino acid sites are indicated by an asterisk (100% similarity) or a dot (90% similarity) below the alignment. The N-terminal signal peptide region and C-terminal cysteine-rich domain are underlined. Boxed amino acids show potential proprotein convertase (PC) cleavage sites, which recognize the consensus motif [R/K]-[R/K], or [R/K]-(X)n-[R/K], where n = 2, 4 or 6.

All SCRiPs were found to consist of a hydrophobic, N-terminal, signal peptide and a C-terminal cysteine-rich domain. A peptide cleavage site is present in all SCRiPs as predicted by SignalIP [13]. In addition, almost all SCRiPs (except for Mfav-SCRiP2 and 5) possess a potential proprotein convertase (PC) cleavage site [14], [15]. We refer to the precursor protein as “preproprotein”, to the processed protein after cleavage of the signal peptide as “proprotein”, and to the product of the PC as “mature protein”. Since Mfav-SCRiP2 and 5 lack a PC cleavage site, the protein is considered “mature” after signal peptide cleavage. The predicted signal peptide is between 19–24 amino acids in length, generating products ranging from 41–48 amino acids after signal and preproprotein cleavage (Table 2). The mature proteins consist of a cysteine-rich domain (Figure 1) with a highly conserved spacing motif of cysteine residues Cx_6–7_Cx_4–6_CPx_3–6_Cx_4–6_Cx_5–6_CCCx_2–5_, where x is any amino acid and the number of residues denoted by indices. The molecular weights of the mature proteins range from 4.3 to 5.8 kD. All SCRiPs are anionic with net charges ranging from −1 to −10 with the exception of Mcap-SCRiP1a and Mcap-SCRiP1b (both +1) (Table 2). Mfav-SCRiP-encoding sequences could be retraced to development stage-specific EST libraries and were found to be exclusively originating from adult colonies (see [3]).

**Table 2:** Physicochemical properties^a^ of SCRiPs.

SCRiP	Preproprotein	Proprotein	Mature protein	Mature protein
	aa (MW)	aa (MW)	aa (MW)	IP / DE / RK (net)
Mfav-SCRiP1^b^	79 (8,451)	58 (6,203)	41 (4,316)	3.89 / 2 / 0 (−2)
Mfav-SCRiP2^c^	68 (7,769)	-	44 (5,219)	3.80 / 11 / 1 (−10)
Mfav-SCRiP4	81 (8,954)	58 (6,642)	48 (5,464)	3.99 / 8 / 2 (−6)
Mfav-SCRiP5^c^	68 (7,735)	-	44 (5,184)	3.89 / 8 / 2 (−6)
Mfav-SCRiP6	81 (9,330)	58 (7,019)	47 (5,761)	3.99 / 11 / 3 (−8)
Mfav-SCRiP8	74 (8,107)	53 (5,869)	41 (4,380)	5.53 / 4 / 3 (−1)
Amil-SCRiP2	79 (8,907)	58 (6,675)	42 (4,756)	5.15 / 5 / 2 (−3)
Amil-SCRiP3	83 (9,222)	62 (7,041)	42 (4,531)	6.02 / 3 / 2 (−1)
Mcap-SCRiP1a^d^	81 (8,919)	62 (6,893)	40 (4,326)	7.71 / 2 / 3 (+1)
Mcap-SCRiP1b^d^	81 (8,892)	62 (6,889)	40 (4,326)	7.71 / 2 / 3 (+1)

a Number of amino acids (aa), molecular weight (MW), isoelectric point (IP), number of negative (DE) and positive amino acids (RK), and net-charge (net) of amino acid residues are shown for SCRiP members identified in this study. In addition to aa and MW for the complete protein (preproprotein), data are shown for both the proprotein, i.e. the product after cleavage of the signal peptide and the mature protein, i.e. the product after proprotein convertase processing. D – aspartate, E – glutamate, R – arginine, K – lysine.

b only sequence with β-defensin motif.

c no proprotein convertase cleavage site.

d mature proteins are identical.

### Phylogenetic analysis

**Figure 2:**
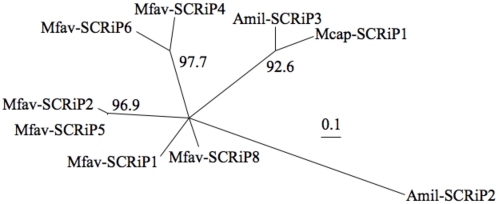
Un-rooted maximum likelihood tree of SCRiP sequences identified in this study. The un-rooted maximum likelihood tree was constructed using the proposed general time reversible (GTR) nucleotide substitution model with discrete gamma. Bootstrap replicate (n = 1000) recovery rates are shown at the internal nodes, when clusters were supported at a level of >80%. Scale = nucleotide substitutions per site.

Multiple sequence alignments of SCRiP sequences displayed a high degree of diversity except for the conserved cysteine sites. In order to increase the quality of the alignment, both manual curation and exclusion of gaps columns were performed prior to phylogenetic reconstruction. The resulting maximum likelihood tree supports the grouping of Mfav-SCRiP2 and Mfav-SCRiP5, Mfav-SCRiP4 and Mfav-SCRiP6, and Mcap-SCRiP1 and Amil-SCRiP3, while the relationship between the remaining SCRiPs, i.e. Mfav-SCRiP1, 8 and Amil-SCRiP2 could not be resolved (Figure 2). These results suggest that Mfav-SCRiP2/5 and Mfav-SCRiP4/6 arose through gene duplication within *M. faveolata*. The Mcap-SCRiP1 and Amil-SCRiP3 pair consists of SCRiPs from different species.

### Gene expression data

**Figure 3:**
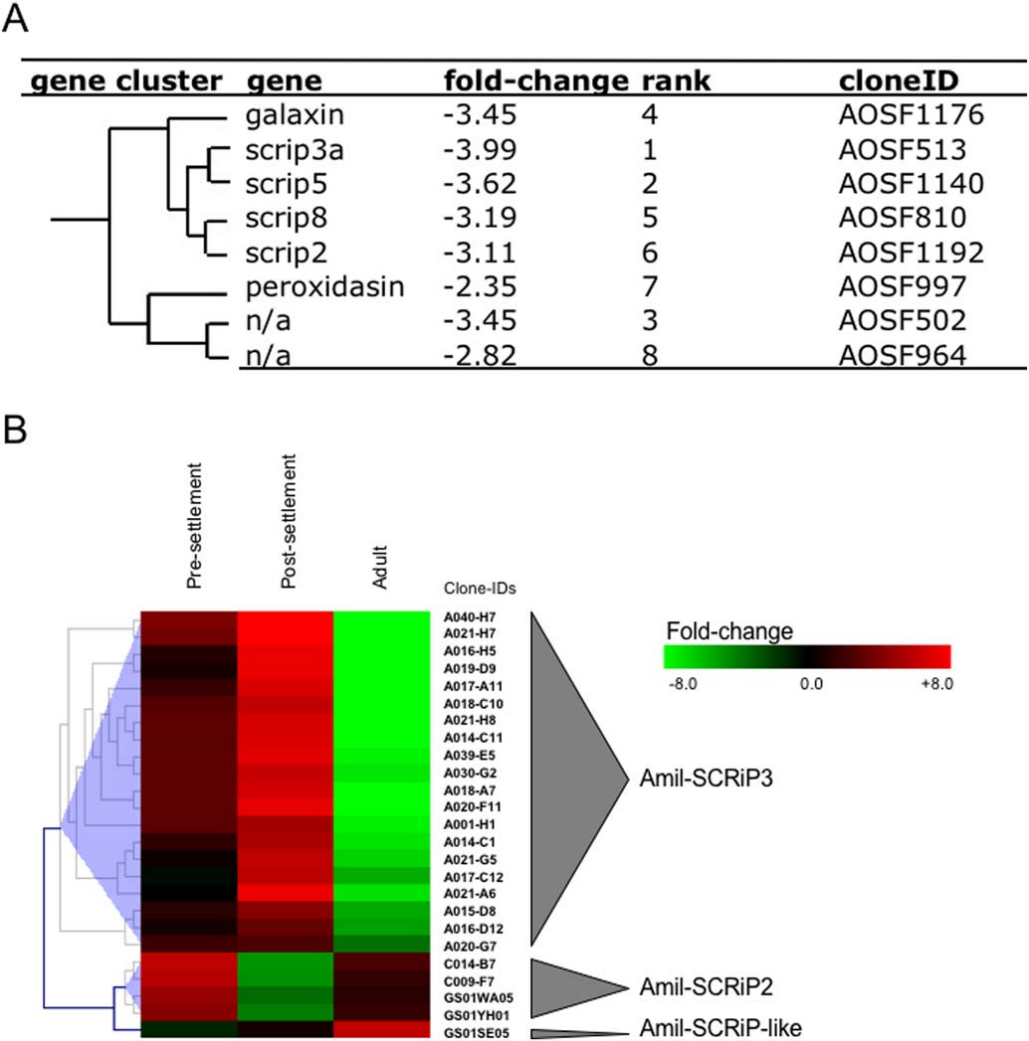
Gene expression microarray data of SCRiPs in thermally stressed *Montastraea faveolata* (A) and different developmental stages in *Acropora millepora* (B). (A) Cluster of most down-regulated genes in thermally stressed *M. faveolata* showing fold changes, rank of most down-regulated genes, and clone IDs according to data available under GEO accession: GSE10630. (B) Heat map of log_2_-transformed signal intensities of Amil-SCRiP gene expression shown as ratios of successive developmental stages. Pre-settlement = ratio of pre-settlment over prawn chip; Post-settlement = ratio of post-settlement over pre-settlement; Adult = ratio of adult over post-settlement. CloneID annotations are available under GEO accession: GSE11251. Note that same numbering does not imply orthology between SCRiPs from different coral species.

Previously, we assayed the expression of 1,310 genes in heat-stressed versus non-stressed *M. faveolata* fragments [10]. Hierarchical clustering of 309 differentially expressed genes, grouped together 4 Mfav-SCRiP genes (Mfav-SCRiP3a, 5, 8 and 2), galaxin, peroxidasin and two non-annotated genes. All genes within this cluster were highly down-regulated upon exposure to heat-stress, ranking positions 1–8 in terms of fold-changes, which ranged from −3.99 to −2.35 (Figure 3A). Expression of the Mfav-SCRiP1 gene was less strongly down-regulated (−1.21), while the fold-changes of Mfav-SCRiP4 and 6 were statistically non-significant.

A recent study investigated transcriptomic patterns in different developmental stages in the coral *Acropora millepora* [11]. We identified the cDNA sequences corresponding to SCRiP sequences by tBLASTx searches, and were interested in the patterns of differentially expressed Amil-SCRiPs (Figure 3B). Both Amil-SCRiP2 and Amil-SCRiP3 experienced an increase in expression levels from the prawnchip to pre-settlement stage (fold-change higher in Amil-SCRiP2), but then showed opposite patterns when proceeding to post-settlement (Amil-SCRiP2: down, Amil-SCRiP3: up) and adult stages (Amil-SCRiP2: up, Amil-SCRiP3: down). A third Amil-SCRiP-like sequence was also identified, which showed yet a different pattern compared to Amil-SCRiP2 and Amil-SCRiP3, i.e. a strong increase only in the adult life stage of development.

## Discussion

### *In silico* discovery of SCRiPs

Genomic resources for symbiotic, reef-building corals as well as symbiotic and non-symbiotic sea anemones have or will soon become available [2]–[5]. We can therefore expect new insight into the evolutionary history and mechanistic processes of coral genes involved in 1) the symbiotic lifestyle and 2) the ability to calcify, among other aspects specific to coral biology. In benefiting from the availability of EST data from a number of reef-building coral species and the genome project for the sea anemone *Nematostella vectensis* [2], we identified a gene family that is taxonomically restricted to scleractinian corals.

The discovery of SCRiPs and the characterization of the novel cysteine-rich motif required a combinatorial data mining approach, i.e. an alternation of tBLASTn and HMM guided searches. Without this iterative search strategy, we would have detected only 4 (tBLASTn only) or 6 (HMM query) out of the 9 sequences now reported for *M. faveolata*. We suggest that this strategy of data mining is not only useful, but also indispensable, when the discovery of homologous sequences in a given dataset is in need of optimization. The power of this search strategy has previously been demonstrated in a similar search for β-defensins that led to the discovery of 28 new human and 43 new mouse β-defensin sequences [16]. Given the limited size of scleractinian EST databases, we suggest that this approach sets the stage for the discovery of additional homologous sequences, and possibly other protein families when more genomic data become available.

### Putative physiological role of SCRiPs

Although our initial search was designed to detect similarities with β-defensins, there is broad support that the functional role of SCRiPs is unlikely to resemble those of typical antimicrobial peptides. First, an InterproScan detected a β-defensin domain (PF00711) only in Mfav-SCRiP1. Second, SCRiPs cover a wide range of net charges, including highly anionic proteins, a feature that is not in concordance with typical antimicrobial peptides, which are generally positively charged (for a review see [17]). Finally, despite the availability of a hexacorallian genome (*N. vectensis*), SCRiPs are only found in reef-building corals, lending support to the idea that this gene-family arose in accordance to adaptations that are specific to that lineage.

For model organisms, a variety of molecular and cell-biological methods are available and readily applied to study the function of unknown genes. For corals, however, it is important to understand that the lack of such tools impedes any inferences on the function of SCRiPs, so that we currently depend on systematic reasoning based on the data available to discuss their potential role. Interestingly, the gene expression data for *M. faveolata* showed that some SCRiP family members were among the most down-regulated genes as a response to thermal stress (Figure 3A), a condition that is known to slow down the rate of calcification in corals. Furthermore, they grouped into the same gene-expression cluster with galaxin, which was identified by Fukuda *et al.* (2003) as a major component of the calcifying organic matrix in the coral *Galaxia fascicularis* [18], [19]. Given this scenario, some molecular features might support the involvement of SCRiPs in biomineralization such as: (a) the presence of a signal peptide region (Figure 1), (b) the fact that Mfav-SCRiP transcripts were restricted to cDNA libraries of adult colonies, (c) low pI values (as low as 3.80) and negative net-charges due to acidic amino acid residues (Table 2), and (d) a cysteine-rich mature protein (Figure 1) that could interact via disulfide-bonds with other cysteine-rich organic matrix molecules like galaxin. On the other hand, the expression patterns observed in different life history stages of *A. millepora* seem to contradict the idea that SCRiPs may be involved in the formation of the coral skeleton. Given the assumption that SCRiPs play similar physiological roles, we would expect a consistent up-regulation in calcifying, i.e. in post-settlement and adult, life-stages. Instead, different SCRiP members appear to have distinct functions. Based on these data, it appears plausible that SCRiPs evolved linage-specific, and subsequently acquired similar, but different (sub-functionalization), or new (neo-functionalization) functions after gene-duplication events [20].

### Evolution of the SCRiP gene family

The abundance of SCRiP genes and their identification in different coral species, from highly divergent coral lineages (i.e. Robust and Complex clades [21], [22]) suggest an early origin within Scleractinia. Our results suggest that SCRiPs are missing in the genome of *N. vectensis*, while gene duplication events must have occurred to generate the observed diversity in SCRiPs in corals. The phylogenetic analysis suggests that the short branch lengths of both the Mfav-SCRiP2/5 and Mfav-SCRiP4/6 cluster (Figure 2) are the likely result of recent taxon-specific duplication events. It will be interesting to explore the genomic organization of SCRiP loci to examine whether lineage-specific tandem duplications may have occurred in *M. faveolata*.

For the clustering of Mcap-SCRiP1 and Amil-SCRiP3, true orthology is likely to be the best explanation, while the resolution of the remaining SCRiPs was constrained by insufficient information (soft polytomy). Although there is disparity in the sequence data sets analyzed (*M. faveolata*: 3,837 ESTs, *A. millepora*: 10,247 ESTs) and it is premature to infer a larger gene family expansion within *M. faveolata*, it is intriguing to note the higher number of *M. faveolata* SCRiPs relative to *A. millepora*.

Early models for the evolution of duplicated genes [23] suggested that one copy will be subject to mutational decay, unless it acquires a new beneficial function [24], while the other one retains its original function. However, in some cases, the maintenance of multiple gene copies can be evolutionarily favorable [25]. For example, lineage-specific expansion (LSE) of genes may be driven when large quantities of a gene product are beneficial for an organism's morphological structures [26]. In our case, SCRiPs may have originally evolved or been acquired after the branching of scleractinians, and subsequently undergone LSE. According to the model of birth-and-death evolution, some of the duplicated genes may diverge, but retain functionality, while other copies may become pseudogenes [27].

### Future outlook

In order to better understand the role of SCRiPs, future work addressing the function and evolution of these genes is necessary. Follow-up experiments will encompass 1) the localization of SCRiP gene expression by whole-mount *in situ* hybridization; and 2) functional characterization after heterologous over-expression and isolation of mature SCRiPs. Finally, it will be insightful to understand the chromosomal context of SCRiPs when coral genome data become available.

## Materials and Methods

### Expressed sequence tag data and nucleotide sequence databases

We used previously published EST data from the coral *Montastraea faveolata* and the sea anemone *Aiptasia pallida* [5]. In addition, we used NCBI's nt, nr and est_others databases as well as databases provided by the Department of Energy Joint Genome Institute (JGI) for *Nematostella vectensis* available at http://genome.jgi-psf.org/Nemve1/Nemve1.home.html [2].

### Isolation of nucleic acids and sequencing of SCRiP cDNA sequences

Genomic DNA was isolated from *M. faveolata* sperm using a modified bead-beat protocol. Briefly, coral sperm embedded in 2% agarose was bead-beaten (0.5 mm zirconia/silica beads) in equal volumes of DNA extraction buffer and phenol∶chloroform∶isoamylalcohol (25∶24∶1). After a second organic extraction, the DNA was obtained by standard ethanol precipitation. For the extraction of RNA, Qiazol and the RNeasy Mini colums (Qiagen, Hilden, Germany) were used according to the manufacturer's protocols. One modification to the protocols involved the pulverization of frozen coral samples on dry ice using a mortar and pestle prior to sample processing. The RNA was reverse-transcribed using the SuperScriptIII RT transcriptase-kit (Invitrogen, Carlsbad, CA, USA). We confirmed the sequence of Mfav-SCRiP1 to be coral-derived by PCR analysis [94°C – 1 min; 94°C – 30 s, 56°C – 30 s, 72°C – 1 min (35×), REDgDNA *Taq* Polymerase (SigmaAldrich, St. Louis, MO)] using DNA of different origins and subsequent sequencing. A 473 bp and 923 bp long fragment of the Mfav-SCRiP1 sequence was amplified with the primers Mfav-SCRiP1_fw and Mfav-SCRiP1_rv using cDNA or genomic DNA as a template (Figure S2). Sequencing of the PCR product derived from genomic DNA was performed in duplicate at the UC Berkeley DNA Sequencing Facility. Primer sequences (Table S1) were designed using Primer3 [28]. Rapid amplification of cDNA 3′ and 5′ ends (3′/5′RACE), subsequent cloning and sequencing was done according to the manufacturer's instructions (GeneRacer and TOPO-TA cloning kit for sequencing; Invitrogen, Carlsbad, CA, USA). Accession numbers of sequences described in this study are shown in Table 3.

**Table 3:** Summary of SCRiP sequences identified in this study.

SCRiP	Evidence	Accession numbers
Mfav-SCRiP1	7 cDNA clones, gDNA	BK006525
Mfav-SCRiP2	1 cDNA clone, RACE clones	BK006526
Mfav-SCRiP3a	1 cDNA clone	BK006527
Mfav-SCRiP3b	1 cDNA clone	BK006528
Mfav-SCRiP4	3 cDNA clones	BK006529
Mfav-SCRiP5	1 cDNA clone, 4 RACE clones	BK006530
Mfav-SCRiP6	5 cDNA clones	BK006531
Mfav-SCRiP7	1 cDNA clone and RACE clones	BK006532
Mfav-SCRiP8	1 cDNA clone and RACE clones	BK006533
Amil-SCRiP1	single EST read	BK006534
Amil-SCRiP2	6 cDNA clones	BK006535
Amil-SCRiP3	27 cDNA clones	BK006536
Mcap-SCRiP1a	GenBank entry	BK006537
Mcap-SCRiP1b	GenBank entry	BK006538

### Hidden Markov Model and BLAST searches

Sequences containing the conserved six-cysteine motif of β-defensins, known antimicrobial peptides in other animals, were obtained from the conserved domain database (pfam00711) of the National Center for Biotechnology Institute (NCBI; http://www.ncbi.nlm.nih.gov/Structure/cdd/cdd.shtml) and aligned using the ClustalW software [29]. The multiple sequence alignment served as an input file for the software package HMMER (Version 2.3.2; http://hmmer.wustl.edu) to construct and calibrate a model for β-defensins using the tools HMMBUILD and HMMCALIBRATE, respectively. The resulting model was queried against a sixframe-translated assembly using CAP3 [30] of *M. faveolata* ESTs [5]. Subsequently, HMM models for SCRiPs were iteratively built by using the first discovered SCRiP as an initial model and appending newly discovered proteins to form multiple sequence alignments upon which the models were built as described above. Similarity searches of nucleotide and amino acid sequences in downloaded GenBank and other databases (SymBioSys: [31]) were performed using BLAST [32].

### Nucleotide and amino acid sequence analyses

EST and RACE sequences were analyzed and consensus sequences generated using the software BioEdit [33]. Signal peptide cleavage sites were detected using neural networks (NN) and Hidden Markov Models (HMM) trained on eukaryotes using the online-tool SignalP (http://www.cbs.dtu.dk/services/SignalP; [13]). Physicochemical properties of the proteins were calculated using ProtParam [34].

### Multiple sequence alignment and Maximum Likelihood gene tree construction

For multiple sequence alignments, we used only sequences for which multiple sources of evidence were available, i.e. EST or RACE reads, except for SCRiPs found in the coral *Montipora capitata* (Mcap-SCRiP1a and Mcap-SCRiP1b), for which we assumed the EST reads to represent either isoforms or to contain a few sequencing errors. For this reason, we used only one Mcap-SCRiP sequence (Mcap-SCRiP1) for multiple sequence alignment and gene tree construction. The alignment was done using the software T-Coffee [35]. For gene tree construction, alignment positions with gaps were removed, before amino acids were back-translated. The resulting nucleotide sequence alignment was manually curated and the software Treefinder [36] used for phylogenetic analyses. The general time reversible (GTR) model of sequence evolution with discrete gamma was suggested and used to build a consensus maximum likelihood tree after 10,000 bootstrap replicates. The resulting gene topology was visualized in TreeView [37].

### Gene expression microarray data and analysis

Gene expression data for the Mfav-SCRiPs originate from a heat-stress experiment (Gene Expression Omnibus GSE10630) described in [10]. Briefly, one colony of *M. faveolata* was collected, fragmented and divided into a control and a treatment tank. After an acclimation period of 3 days, the treatment tank temperature was increased by 3°C for 257 h before the fragments were frozen in liquid nitrogen and transported to the laboratory for molecular analysis. Samples were processed for cDNA microarray experiments (1,310 features) as described in DeSalvo et al. (2008), and differentially expressed genes were determined using J/MAANOVA [38] and the Empirical-Bayes Fs statistic [39]. Differentially expressed genes were hierarchically clustered using the TM4 Microarray Software Suite [40] with Euclidian distance metric and average linkage method. In addition, we utilized gene expression microarray data (Gene Expression Omnibus GSE11251) to illustrate the gene expression patterns for Amil-SCRiPs in different developmental stages [11].

## Supporting Information

Figure S1 **Multiple sequence alignment of SCRiP3a and SCRiP3b.** Perfect matches with Mfav-SCRiP2 are shown in boldface.
(0.04 MB DOC)

Figure S2 **Confirmation of origin of Mfav-SCRiP1 by PCR analysis.** Amplification of Mfav-SCRiP1 yields discrete bands when genomic DNA (with intron), or cDNA (intronless) were used as templates. 18SrDNA specific primers were used as positive control for PCR reactions. The 1,100 bp band was sequenced and confirmed to originate from Mfav-SCRiP1. Mf = *Montastraea faveolata*; Sym = *Symbiodinium* spp.; gDNA = genomic DNA; cDNA = comlementary DNA.
(0.13 MB DOC)

Table S1 Primer sequences used in this study.
(0.03 MB DOC)
