# Supplementary material for: Identification and Gene Expression Analysis of a Taxonomically Restricted Cysteine-Rich Protein Family in Reef-Building Corals
Source: PLoS One. 2009 Mar 13;4(3):e4865. doi: 10.1371/journal.pone.0004865 (PMC2652719; doi:10.1371/journal.pone.0004865)
Supplement: Figure S1 — Multiple sequence alignment of SCRiP3a and SCRiP3b. Perfect matches with Mfav-SCRiP2 are shown in boldface. (0.04 MB DOC) [file pone.0004865.s001.doc]

>Mfav-SCRiP3a

**MAVKFHLCLLLIIVVGMGAHVAFAD**KPLCDLPHGTCYYYKDPCPDN**MPVDCSE**E**FYCTLETNKCCC**NEPPPTERPLCDLPHGTCYYHKDPCPDNMPVDCSQDFKCTLDTNKCCCYE

>Mfav-SCRiP3b

-----------------------**AD**KPLCDLPHGTCYYYKDPCPDN**MPVDCSE**K**FYCTLETNKCCC**NEPPPTERPLCDLPHGTCYYHKDPCPDNMPVDCSQHFKCTLDTNKCCCY-

>Mfav-SCRiP2

**MAVKFHLCLLLIILVGMGAHVAFAD**QQFCDHPYGTCYYVEDECPED**MPVDCSE**N**FYCTEPTNKCCC**YE------------------------------------------------

**Figure S1. Multiple sequence alignment of SCRiP3a and SCRiP3b.**

Perfect matches with Mfav-SCRiP2 are shown in boldface.
