# Supplementary material for: Identification and Gene Expression Analysis of a Taxonomically Restricted Cysteine-Rich Protein Family in Reef-Building Corals
Source: PLoS One. 2009 Mar 13;4(3):e4865. doi: 10.1371/journal.pone.0004865 (PMC2652719; doi:10.1371/journal.pone.0004865)
Supplement: Figure S2 — Confirmation of origin of Mfav-SCRiP1 by PCR analysis. Amplification of Mfav-SCRiP1 yields discrete bands when genomic DNA (with intron), or cDNA (intronless) were used as templates. 18SrDNA specific primers were used as positive control for PCR reactions. The 1,100 bp band was sequenced and confirmed to originate from Mfav-SCRiP1. Mf = Montastraea faveolata; Sym = Symbiodinium spp.; gDNA = genomic DNA; cDNA = comlementary DNA. (0.13 MB DOC) [file pone.0004865.s002.doc]

*
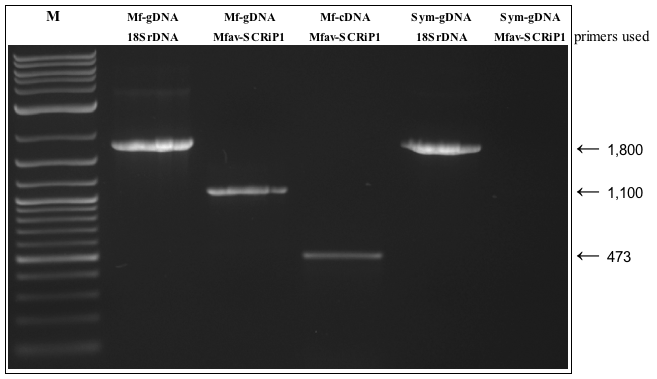
*

**Figure S2. Confirmation of origin of Mfav-SCRiP1 by PCR analysis.**

Amplification of Mfav-SCRiP1 yields discrete bands when genomic DNA (with intron), or cDNA (intronless) were used as templates. 18SrDNA specific primers were used as positive control for PCR reactions. The 1,100 bp band was sequenced and confirmed to originate from Mfav-SCRiP1. Mf=*Montastraea faveolata*; Sym=*Symbiodinium* spp.; gDNA=genomic DNA; cDNA=comlementary DNA.
