# Supplementary material for: Identification and Gene Expression Analysis of a Taxonomically Restricted Cysteine-Rich Protein Family in Reef-Building Corals
Source: PLoS One. 2009 Mar 13;4(3):e4865. doi: 10.1371/journal.pone.0004865 (PMC2652719; doi:10.1371/journal.pone.0004865)
Supplement: Table S1 — Primer sequences used in this study. (0.03 MB DOC) [file pone.0004865.s003.doc]

Table S1. Primer sequences used in this study.

| SCRiP | Primer sequence (5’-3’) |
| --- | --- |
| Mfav-SCRiP1_fw: | ATGGAAGCTAAGTTTCGTTTGAG |
| Mfav-SCRiP1_rv: | TCTCGTTAATTTTGTCCCCAAT |
| Mfav-SCRiP2_3’RACE: | CCGGTTGACTGTTCAGAAAATTT |
| Mfav-SCRiP2_5’RACE: | CATCACCTCGCATCATTCGT |
| Mfav-SCRiP5_3'RACE: | TGCAAGCTAAGTTCAAATTAAACATGGC |
| Mfav-SCRiP5_5’RACE: | GCTTTTATTCCACTGCTTCAAGTGC |
| Mfav-SCRiP7_3’RACE: | GTTTGCATGCCCGATTGG |
| Mfav-SCRiP7_5’RACE: | CAGCAGCAACGACGGTTGT |
| Mfav-SCRiP8_3’RACE: | CCCAGGGAAGCCTCATTTC |
| Mfav-SCRiP8_5’RACE: | CCATTGATCGAAGCACAATCC |
